# Ethiopian Medicinal Plants Traditionally Used for the Treatment of Cancer, Part 2: A Review on Cytotoxic, Antiproliferative, and Antitumor Phytochemicals, and Future Perspective

**DOI:** 10.3390/molecules25174032

**Published:** 2020-09-03

**Authors:** Solomon Tesfaye, Kaleab Asres, Ermias Lulekal, Yonatan Alebachew, Eyael Tewelde, Mallika Kumarihamy, Ilias Muhammad

**Affiliations:** 1 School of Pharmacy, College of Health Sciences, Addis Ababa University, Churchill Street, 1176 Addis Ababa, Ethiopia; kaleab.asres@aau.edu.et (K.A.); yonalebachew@gmail.com (Y.A.); eyaeltd@gmail.com (E.T.); 2 Department of Plant Biology and Biodiversity Management, College of Natural and Computational Sciences, The National Herbarium, Addis Ababa University, 34731 Addis Ababa, Ethiopia; ermias.lulekalm@aau.edu.et; 3 National Center for Natural Products Research, Research Institute of Pharmaceutical Sciences, School of Pharmacy, University of Mississippi, University, MS 38677, USA; mkumarih@olemiss.edu

**Keywords:** medicinal plants, cancer, Ethiopia, phytochemistry

## Abstract

This review provides an overview on the active phytochemical constituents of medicinal plants that are traditionally used to manage cancer in Ethiopia. A total of 119 articles published between 1968 and 2020 have been reviewed, using scientific search engines such as ScienceDirect, PubMed, and Google Scholar. Twenty-seven medicinal plant species that belong to eighteen families are documented along with their botanical sources, potential active constituents, and in vitro and in vivo activities against various cancer cells. The review is compiled and discusses the potential anticancer, antiproliferative, and cytotoxic agents based on the types of secondary metabolites, such as terpenoids, phenolic compounds, alkaloids, steroids, and lignans. Among the anticancer secondary metabolites reported in this review, only few have been isolated from plants that are originated and collected in Ethiopia, and the majority of compounds are reported from plants belonging to different areas of the world. Thus, based on the available bioactivity reports, extensive and more elaborate ethnopharmacology-based bioassay-guided studies have to be conducted on selected traditionally claimed Ethiopian anticancer plants, which inherited from a unique and diverse landscape, with the aim of opening a way forward to conduct anticancer drug discovery program.

## 1. Introduction

Cancer is a major global health challenge that affects millions of people annually across the world. Recent estimates showed about 18.1 million new cases of cancer and 9.6 million cancer-related deaths worldwide [1]. Moreover, due to population growth, aging, and increased prevalence of key risk factors, this figure is expected to rise in the coming years. According to the same report, different from other parts of the world, cancer death (7.3%) is higher than cancer incidence (5.2%) in Africa. This is mainly attributed to lack of adequate health care facilities as well as professionals, lack of early cancer detection system, and poor access to chemotherapeutic treatments. Due to these and other factors, including socio-economic conditions, the majority of the population of Africa has relied on traditionally used medicinal herbs and/or plants as a monotherapy or in combination with clinically approved anticancer drugs.

Medicinal plants have been a rich source of clinically effective anticancer agents for the past few decades. Over 60% of the currently used anticancer drugs are either directly derived from plants or inspired by their novel phytochemicals [2] and/or unique ligands as secondary metabolites. In spite of such success, the importance of medicinal plants as a source of leads for anticancer drug discovery was marginalized in comparison with other advanced approaches. This could be due to issues associated with intellectual property rights and securing not enough amounts of plant material which results in the slowness of working with natural products [3]. However, despite these drawbacks, medicinal plant-based drug discovery and development has made a comeback to find potent and affordable natural products with a new mechanism of action and better toxicological profile due to structural diversity of natural product small molecules (NPSM). For instance, among small molecules approved for cancer treatment between 1940 and 2014, 49% are derived and/or originated from natural products [4].

Ethiopia inherited a unique array of fascinating flora from its diverse landscape. Due to the geographical location and diversity, which favors the existence of different habitat and vegetation zones, Ethiopia is home to a variety of plant species. The Ethiopian flora is estimated to contain 6027 species of higher plants of which more than 10% are estimated to be endemic [5]. Different authors have compiled ethnobotanical and ethnopharmacological profiles and reviews of Ethiopian traditionally used medicinal plants [6,7]. However, published reports regarding isolated bioactive compounds of traditionally used Ethiopean medicinal plants, especially those with cytotoxic properties are scant. However, investigations conducted on plants with cytotoxic properties out side Ethiopia, include the study on *Catha edulis* Forsk [8,9], *Artemisia annua* L., *Rumex abyssinicus* Jacq. [9]., *Carissa spinarum* L., *Dodonaea angustifolia* L.f., *Jasminum abyssinicum* Hochst. ex DC., *Rumex nepalensis* Spreng., *Rubus steudneri* Schweinf. and *Verbascum sinaiticum* Benth. [10], *Viola abyssinica* Steud. ex Oliv. [11], *Xanthium strumarium* L. [12], *Senna singueana* (Del). Lock [13], *Glinus lotoides* L. [14], *Kniphofia foliosa* Hochst [15], *Sideroxylon oxyacanthum* Baill., *Clematis simensis* Fresen, and *Dovyalis abyssinica* (A. Rich) Warburg [16]. Thus, for further evaluation, identification, or modification of anticancer leads, thorough review of the chemistry and pharmacology of medicinal plants from relatively uncovered traditional medical systems is crucial. Therefore, in continuation of our previous mini-review [17], in which we documented both ethnobotanical and ethnopharmacological evidence of Ethiopian anticancer plants involving mostly the cytotoxic and antioxidant activities of crude extracts, here, in this review, we comprehensively document the cytotoxic and antiproliferative constituents from anticancer plants those traditionally used in Ethiopia. The secondary metabolites reported from each medicinal plant species are categorized based on the class of natural products they belong to.

## 2. Traditional Uses of Selected Plants

**Figure 1:**
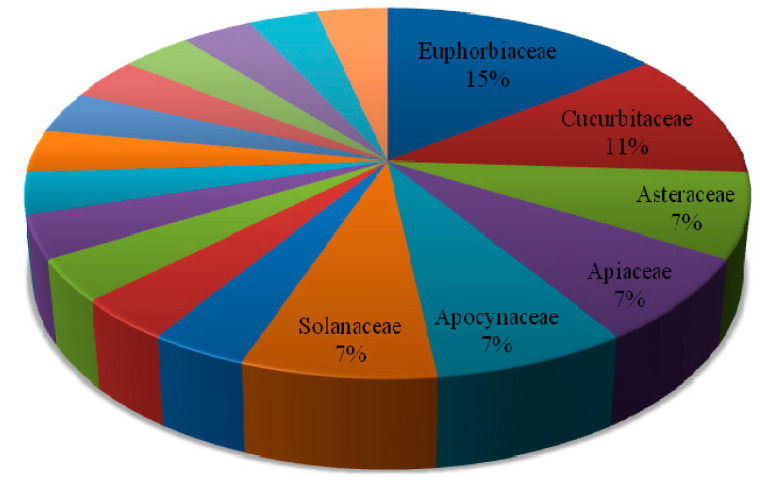
Major plant families (in %) of reviewed plants species vegetation zone of Ethiopia [18] (the unmarked blocks are other species).

A total of 27 anticancer traditional medicinal plants that belong to 18 botanical families and 27 genera are identified in this review. The botanical families Euphorbiaceae and Cucurbitaceae were the most dominant, represented with 15% and 11% of the selected plant species, respectively (Figure 1). All of the reviewed plants have direct traditional uses for treating either ailments with cancer-like symptoms (determined by traditional practitioner) or for laboratory-confirmed cancer cases. Besides treating cancer, the plants selected in this review are also cited for their various traditional uses, including for the treatment of eczema, leprosy, rheumatism, gout, ringworm, diabetes, respiratory complaints, warts, hemorrhoid, syphilis, and skin diseases (Table 1). The output calls for the need for further phytochemical and pharmacological investigation giving priority to those plants which have been cited most for their use to treat cancer.

## 3. Phytochemistry of Ethiopian Anticancer Plants

The present review reports secondary metabolites isolated from 27 plants that are traditionally used to treat different types of cancer in Ethiopia. Phytochemical investigations of traditionally used Ethiopian anticancer plants have led to the isolation of compounds that belong to different classes of natural products [10,57]. In this review, we have not included plants those displayed compounds with very low cytotoxic/antiproliferative activity (i.e., IC_50_ (Concentration that inhibited cell proliferation by 50%)/ED_50_ (Effective dose for 50% of the population) > 50 µg/mL or > 100 µM, in most cases, except few where compounds tested against a panel of cell lines) or plants from which no anticancer compounds were isolated/reported. This review compiled and discussed the potential anticancer/antiproliferative agents based on the types of secondary metabolites, such as terpenoids, phenolic compounds, alkaloids, steroids, and lignans.

### 3.1. Terpenoids

Terpenoids are classified according to the number of their isoprene unit as hemi-, mono-, di-, tri-, tetra-, and polyterpenes [58]. Various studies reported that the anticancer activity of terpenoids is due to the inhibition of inflammation, cancer cell proliferation, angiogenesis and metastasis, and induction of programmed cell death [59]. Triterpenoids are one important class of terpenoids, which contain isopentenyl pyrophosphate oligomers [60]. They are biosynthesized by plants through cyclization of 30-carbon intermediate squalene and include various structural subclasses [61]. Several triterpenoids have been shown to have anticancer activity.

**Figure 2:**
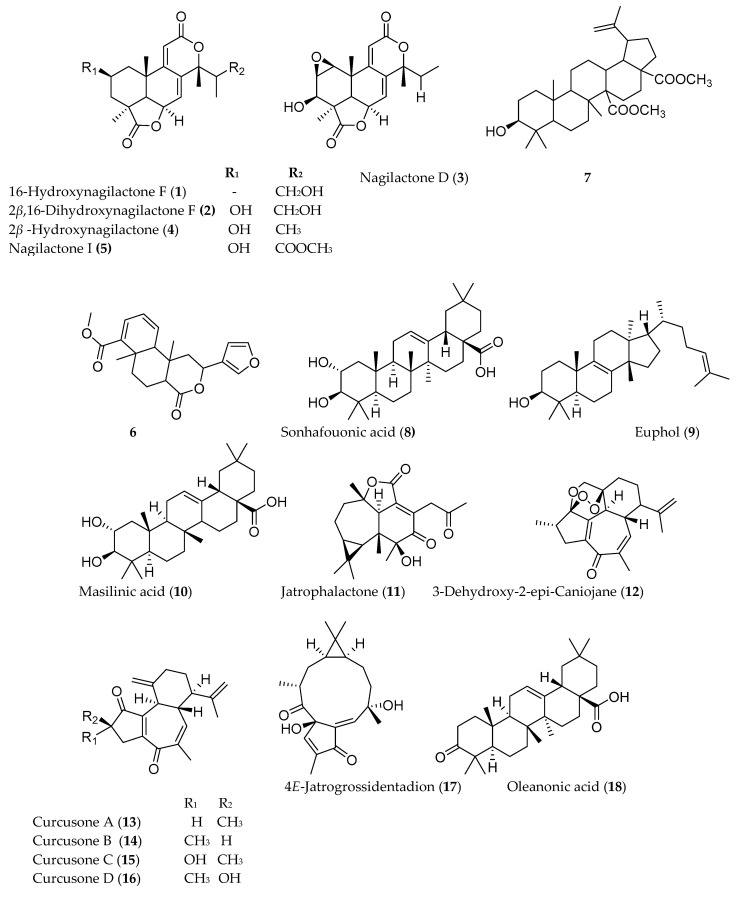

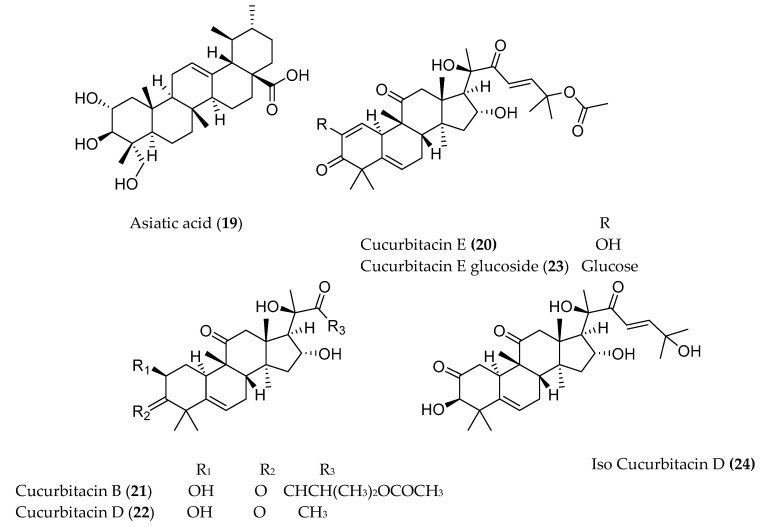
Structures of anticancer terpenoids reported from plants available in Ethiopia.

Among the different types of triterpenoids, pentacyclic triterpnoids display the most potent anti-inflammatory and anticancer activity [62]. Addo et al. [63] reported the isolation of two new nagilactones along with seven known from the root of *Podocarpus falcatus* (Thunb.) collected from Berga forest, Addis Alem, central Ethiopia. *P. falcatus*is traditionally used to treat jaundice, gastritis, and amoeba [6]. Among the isolated compounds 16-hydroxynagilactone F (**1**), 2*β*,16-dihydroxynagilactone F (**2**), 7*β*-hydroxymacrophyllic acid, nagilactone D (**3**), 15-hydroxynagilactone (**4**), and nagilactone I (**5**) (Figure 2) showed potent antiproliferative activity against HT-29 cell line (IC_50_ < 10 µM) (Table 2). *Premna schimperi*, another traditionally used Ethiopian plant, also showed cytotoxic activity against L929, RAW264.7, and SK.N.SH with IC_50_ values of 11 ± 2.3, 10 ± 2.3, and 1.5 ± 0.3 µg/mL, respectively [57]. The methanolic extract of another commonly used Ethiopian plant, *Croton macrostachyus*, was also shown to possess cytotoxic activity against HTC116 cell line [64]. A diterpenoid compound methyl 2-(furan-3-yl)-6*α*,10*β*-dimethy-l4-oxo-2,4,4*α*,5,6,6*α*,10*α*,10*β*-octahydro-1*H*-benzo[f]isochromene-7-carboxylate) (**6**), demonstrated a moderate cytotoxic activity (IC_50_ = 50 µg/mL). The compound was shown to trigger caspase mediated apoptotic cell death. 3*β*-Hydroxylup-20(29)-ene-27,28-dioic acid dimethyl ester (**7**), isolated from root of *Plumbago zeylanica* collected from India, also exhibited anti-proliferative and anti-migration activity against triple-negative breast cancer cell lines at IC_50_ value of 5 µg/mL [65].

Several terpenoids have been isolated from Ethiopian plants that have claims of having anticancer activity, although these plants may have been collected from other sources. For example, sonhafouonic acid (**8**) from *Zehneria scabra*, collected from Cameroon, demonstrated potent cytotoxicity against brine shrimp assay [66], while Lin et al. [67] showed the antiproliferative activity of euphol (**9**), isolated from *Euphorbia tirucalli* from Taiwan against human gastric cancer cells. Euphol selectively promotes apoptosis by mitochondrial-dependent caspase-3 activation and growth arrest through induction of p27kip1 and inhibition of cyclin B1 in human gastric CS12 cancer cells. It also showed a selective and strong cytotoxicity against other groups of human cancer cell lines such as glioblastoma (the most frequent and aggressive type of brain tumor) [67,68]. The molecular mechanism of action of another anticancer triterpenoid, maslinic acid (**10**), isolated from the leaves of *Olea europaea* has been studied, which induced apoptosis in HT29 human colon cancer cells by directly inhibiting the expression of Bcl-2, increasing that of Bax, releasing cytochrome-C from the mitochondria and activating caspase-9 and then caspase-3 [69]. Similarly, the leaf extract of *Ricinus communis* collected from Malta was also reported for its cytotoxicity against several human tumor cells and induction of apoptosis against human breast tumors, SK-MEL-28. The monoterpenoids 1,8-cineole, camphor and α-pinene, and the sesquiterpenoid *β*-caryophyllene, isolated from *R. communis*, also showed cytotoxicity against similar cell lines in a dose-dependent manner [70].

*Jatropha curcas* is a medicinal plant traditionally used to treat a variety of ailments in different parts of the world including Ethiopia [71]. Investigation of *J. curcas*, collected from China, resulted in the isolation of twelve phorbol esters (diterpenoids) including jatrophalactone (**11**), curcusecon A–J, 4-epi-curcusecon E, curcusone E, 3-dehydroxy-2-epi-caniojane (**12**), curcusone A (**13**), curcusone B (**14**), curcusone C (**15**), curcusone D (**16**), jatrogrosidone, 2-epi-jatrogrossidone, and 4*E*-jatrogrossidentadion (**17**) [72]. Most of these compounds showed potent cytotoxicity with IC_50_ values ranging from 0.084 to 20.6 µM against HL-60, SMMC-7721, A-549, MCF-7, SW480, and HEPG2 cell lines [72,73].

The pentacyclic triterpenoid oleanonic acid (**18**), isolated from *Ekebergia capensis* [74], exhibited potent cytotoxic activity against human epithelial type 2 (HEp2) and murine mammary carcinoma (4T1) cell with IC_50_ values of 1.4 and 13.3 µM, respectively. Another pentacyclic triterpenoid, asiatic acid (**19**), isolated from *Centella asiatica*, also showed 80% growth inhibition of human colorectal (SW480), human stomach (SNU668), and murine colorectal adenocarcinoma (CT26) cell lines with IC_50_ values of 20 µg/mL [75]. The fresh fruit of *Cucumis prophetarum* from Saudi Arabia yielded a series of cucurbitacin and analogs (cucurbitacin E (**20**), cucurbitacin B (**21**), cucurbitacin D (**22**), cucurbitacin F 25-*O*-acetate, cucurbitacin E glucoside (**23**), dihydrocucurbitacin D, hexanor-cucurbitacin D, and isocucurbitacin D (**24**)), of which compounds **20**–**24** showed cytotoxic activity against MCF-7, MDA MB 231, A2780, A2780 CP, HepG2, and HCT-116 with IC_50_ values ranging from 1 to 27.3 µM [76].

**Table 2:** Terpenoids isolated from medicinal plants that are traditionally used to treat cancer in Ethiopia.

Plant Family	Class of Compounds	Cell Lines	IC_50_	Pharmacology	Isolated Active Compounds	Reference
*Ferula communis* L. (Apiaceae)	Daucane Sesquiterpene	Jurkat T-cells		Ionotropism	Ferutinin	[34]
*Vernonia amygdalina* Delile (Asteraceae)	Sesquiterpene lactones	KB	-	-	Vernodalin and Vernomygdin	[77]
*Vernonia hymenolepis* A. Rich. (Asteraceae)	Sesquiterpene Dilactone		-	-	Vernolepin	
*Zehneria scabra* (L.F. Sond) (Cucurbitaceae)	Triterpenoid	Brine shrimp	10 μg/mL		Sonhafouonicacid (**8**)	[66]
*Croton macrostachyus* Hochst. ex Delile* (Euphorbiaceae)	Diterpenoid	HCT116	50 µg/mL	Caspase mediated apoptosis	methyl 2-(furan-3-yl)-6*α*,10*β*-dimethy-l4-oxo-2,4,4*α*,5,6,6*α*,10*α*,10*β*--octahydro-1H-benzo[f]isochromene-7-carboxylate)	[64]
*Euphorbia tirucalli* L. (Euphorbiaceae)	Triterpenoid	CS12	12.8 µg/mL	Apoptosis	Euphol (**9**)	[67]
		AGS	14.7 µg/mL			
		MKN45	14.4 µg/mL			
*Ricinus communis* L. (Euphorbiaceae)	Monoterpenoid	SK-MEL-28	21.67 ± 4.74 µg/mL	Appoptosis	1,8-Cineole, camphor, α-pinene, β-Caryophyllene	[70]
		K-562	24.49 ± 1.61 µg/mL			
		COLO 679	20.14 ± 2.99 µg/mL			
		OAW42	13.52 ± 0.20 µg/mL			
		HT-29	19.86 ± 5.94 µg/mL			
		MCF-7	37.87 ± 3.36 µg/mL			
		PBMC	13.55 ± 0.85 µg/mL			
*Jatropha curcas* L. (Euphorbiaceae)	Diterpenoid	HL-60	8.5 µM		Jatrophalactone (**11**)	[72]
		SMMC-7721	20.6 µM			
		A-549	19.7 µM			
		MCF-7	20.1 µM			
		SW480	19.2 µM			
		HL-60	>40 µM		Curcusecon A-J, 4-epi-curcusecon E, Curcusone E	
		SMMC-7721	>40 µM			
		A-549	>40 µM			
		MCF-7	>40 µM			
		SW480	>40 µM			
		HL-60	2.86 µM		3-Dehydroxy-2-epi-Caniojane (**12**)	
		SMMC-7721	3.94 µM			
		A-549	3.49 µM			
		MCF-7	11.69 µM			
		SW480	14.05 µM			
		HL-60	1.63 µM		Curcusone A (**13**)	
		SMMC-7721	3.10 µM			
		A-549	3.35 µM			
		MCF-7	2.47 µM			
		SW480	2.10 µM			
		HL-60	2.64 µM		Curcusone B (**14**)	
		SMMC-7721	3.30 µM			
		A-549	3.88 µM			
		MCF-7	3.14 µM			
		SW480	2.91 µM			
		HL-60	1.36 µM		Curcusone C (**15**)	
		SMMC-7721	2.17 µM			
		A-549	3.88 µM			
		MCF-7	1.61 µM			
		SW480	1.99 µM			
		HL-60	2.81 µM		Curcusone D (**16**)	
		SMMC-7721	3.58 µM			
		A-549	4.70 µM			
		MCF-7	2.77 µM			
		SW480	2.83 µM			
		HL-60	22.80 µM		Jatrogrosidone	
		SMMC-7721	19.49 µM			
		A-549	34.93 µM			
		MCF-7	21.83 µM			
		SW480	20.06 µM			
		HL-60	23.30 µM		2-epi-Jatrogrossidone	
		SMMC-7721	18.36 µM			
		A-549	36.53 µM			
		MCF-7	22.72 µM			
		SW480	21.08 µM			
		HEPG2	0.084 µM		Curcusone C (**15**)	[73]
			0.153 µM		Curcusone D (**16**)	
			0.183 µM		4*E*-Jatrogrossidentadion (**17**)	
*Premna schimperi* Engl.* (Verbenaceae)	Clerodane diterpene	L929	11 ± 2.3 µg/mL	-	(5*R*,8*R*,9*S*, I OR)-12-Oxo-ent-3,13(16)-clerodjen-15-oic acid	[57]
		RAW264.7	10 ± 2.3 µg/mL			
		SK.N.SH	1.5 ± 0.3 µg/mL			
*Ekebergia capensis* Sparrm. (Meliaceae)	Triterpenoids	HEp2	1.4 µM	-	Oleanonic acid (**18**)	[74]
		4T1	13.3 µM			
*Olea europaea* subsp. Cuspidata (Wall. ex. G. Don) Cif. (Oleaceae)	Triterpenoids	HT-29	28.8 ± 0.9 µg/mL	Apoptosis	Maslinic acid (**10**)	[69]
Podocarpus falcatus* (Podocarpaceae)	Terpenoids-Nagilactones (diterpenoids)	HT-29	0.6 ± 0.4 µM		16-Hydroxynagilactone F (**1**)	[63]
			1.1 ± 0.5 µM		2*β*,16-Dihydroxynagilactone F (**2**)	
			0.3 ± 0.1 µM		2*β*-Hydroxynagilactone F	
			>10 µM		7*β*-Hydroxymacrophyllic acid	
			>10 µM		Macrophyllic acid	
			0.9 ± 0.3 µM		Nagilactone D (**3**)	
			5.1 ± 0.8 µM		15-Hydroxynagilactone (**4**)	
			0.5 ± 0.1 µM		Nagilactone I (**5**)	
			>10 µM		Inumakiol D	
			>10 µM		Ponasterone A	
*Cucumis prophetarum* (Cucurbitaceae)	Triterpenoids	MCF-7	7.2 µM		Cucurbitacin E (**20**)	[76]
		MDA MB 231	2.1 µM			
		A2780	5.4 µM			
		A2780 CP	15.9 µM			
		HepG2	3.4 µM			
		HCT-116	3.4 µM			
		MCF-7	16.0 µM		Cucurbitacin B (**21**)	
		MDA MB 231	0.96 µM			
		A2780	7.6 µM			
		A2780 CP	14.2 µM			
		HepG2	1.7 µM			
		HCT-116	1.7 µM			
		MCF-7	47.9 µM		Hexanor-Cucurbitacin D	
		MDA MB 231	12.0 µM			
		A2780	>100 µM			
		A2780 CP	>100 µM			
		HepG2	37.8 µM			
		HCT-116	30.7 µM			
		MCF-7	26.7 µM		Cucurbitacin D (**22**)	
		MDA MB 231	4.0 µM			
		A2780	21.6 µM			
		A2780 CP	6.9 µM			
		HepG2	5.0 µM			
		HCT-116	7.6 µM			
		MCF-7	18.4 µM		Cucurbitacin F 25-O-acetate	
		MDA MB 231	3.4 µM			
		A2780	15.8 µM			
		A2780 CP	15.2 µM			
		HepG2	10.2 µM			
		HCT-116	11.2 µM			
		MDA MB 231	>100 µM		Dihydrocucurbitacin D	
			27.3 µM		Cucurbitacin E glucoside (**23**)	
			1 µM		Isocucurbitacin D (**24**)	
*Centella asiatica*	Triterpenoids	SW480	20 µg/mL (80% growth inhibition)	Growth inhibition and apoptosis	Asiatic Acid (**19**)	[75]
		SNU668				
		CT26				
*Plumbago zeylanica*	Triterpenoids	MDA-MB-231	5 µg/mL	Inhibits proliferation and migration	3*β*-Hydroxylup-20(29)-ene-27,28-dioic acid (**7**)	[65]

Cell lines: HCT116 = Human colorectal carcinoma, CS12 = Human gastric carcinoma, AGS = Human gastric carcinoma, MKN-45 = Human gastric adenocarcinoma, SK-MEL-28 = Human melanoma, K562 = Human myelogenous leukemia, COLO 679 = Human melanoma, OAW42 = Human ovarian carcinoma, HT-29 = Human colorectal adenocarcinoma, MCF-7 = Human breast adenocarcinoma, PBMC = Peripheral blood mononuclear, HL-60 = Human promyelocytic leukemia, SMMC-7721 = Human hepatocarcinoma, A-549 = Human lung adenocarcinoma, SW480 = Human colorectal, HepG2 = Liver hepatocarcinoma, L929 = Murine fibroblast, RAW264.7 = murine macrophage, SK.N.SH = Human neuroblastoma, HEp-2 = Human epithelial type 2, 4T1 = Murine mammary carcinoma, HT-29 = Human colorectal adenocarcinoma, Caco-2 = Human colon carcinoma, MDA MB 231 = Triple-negative breast cancer, A2780 = Human ovarian carcinoma, A2780 CP = cisplatin-resistant ovarian carcinoma, HCT116 = Human colorectal carcinoma. IC_50_ = Concentration that inhibited cell proliferation by 50%. * Plant material collected from Ethiopi.

### 3.2. Phenolic Compounds

**Figure 3:**
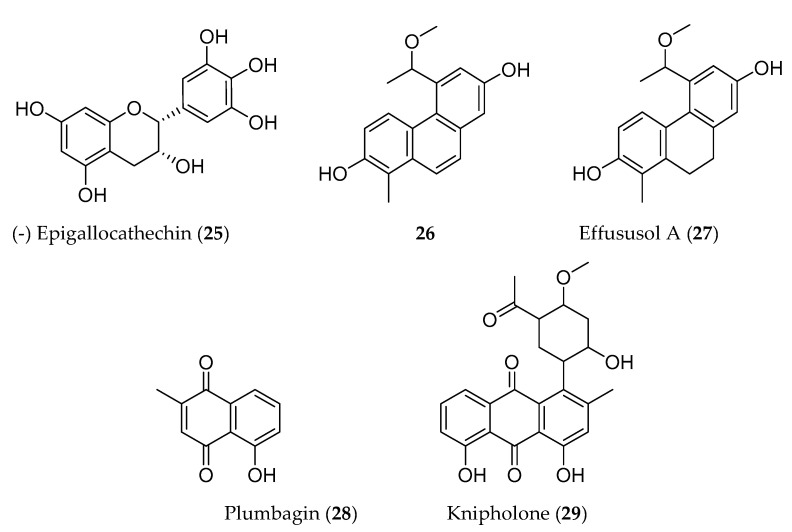
Structures of anticancer phenolic compounds reported from plants available in Ethiopia.

Phenolic compounds are biosynthesized by plants through shikimate, phenylpropanoid, and flavonoid pathways, and have an aromatic ring bearing one or more hydroxyl groups. These compounds have been reported for their antioxidant, antiproliferative, and cytotoxic properties [78]. Many phenolic compounds have been identified elsewhere from the same medicinal plants that are traditionally used to manage cancer in Ethiopia. For instance, (−)-epigallocathechin (**25**) isolated from *Maytenus senegalensis* has showed potent cytotoxic activity against mouse lymphoma cell line (L5178Y) [79]. Likewise, a series phenanthrenes (5-(1-methoxyethyl)-1-methyl-phenanthren-2,7-diol (**26**); effususol A; effusol; dehydroeffusol; dehydroeffusal; 2,7-dihydroxy-1,8-dimethyl-5-vinyl-9,10-dihydrophenanthrene and juncusol; dehydrojuncusol and 1-methylpyrene-2,7-diol) from *Juncus effuses* inhibited the proliferation of five human cancer cell lines (Table 3). Among these, 5-(1-methoxyethyl)-1-methyl-phenanthren-2,7-diol (**26**) (Figure 3) was tested against MCF-7 cancer cell line and showed better cytotoxic activity [80] than all isolated compounds from *J. effuses*. Another group of phenanthrenoids (effususol A, **27**) has also demonstrated potent cytotoxicity against HT-22 cell by inducing caspase-3-mediated apoptosis [81]. Plumbagin (**28**), a naphthoquinone isolated from *Plumbago zeylanica* also induced apoptosis in human non-small cell lung (IC_50_ = 6.1–10.3 µM) [82] and human pancreatic (IC_50_ = 2.1 µM) [83] cancer cell lines. On the other hand, knipholone (**29**) isolated from *Kniphofia foliosa* Hochst collected from Ethiopia, induced necrotic death in mouse melanoma (B16), mouse macrophage tumor (RAW 264.7), human acute monocytic (THP-1), and promonocytic leukaemic (U937) cell lines with IC_50_ values that range from 0.5 ± 0.05 to 3.3 ± 0.39 µM [15].

### 3.3. Alkaloids

**Figure 4:**
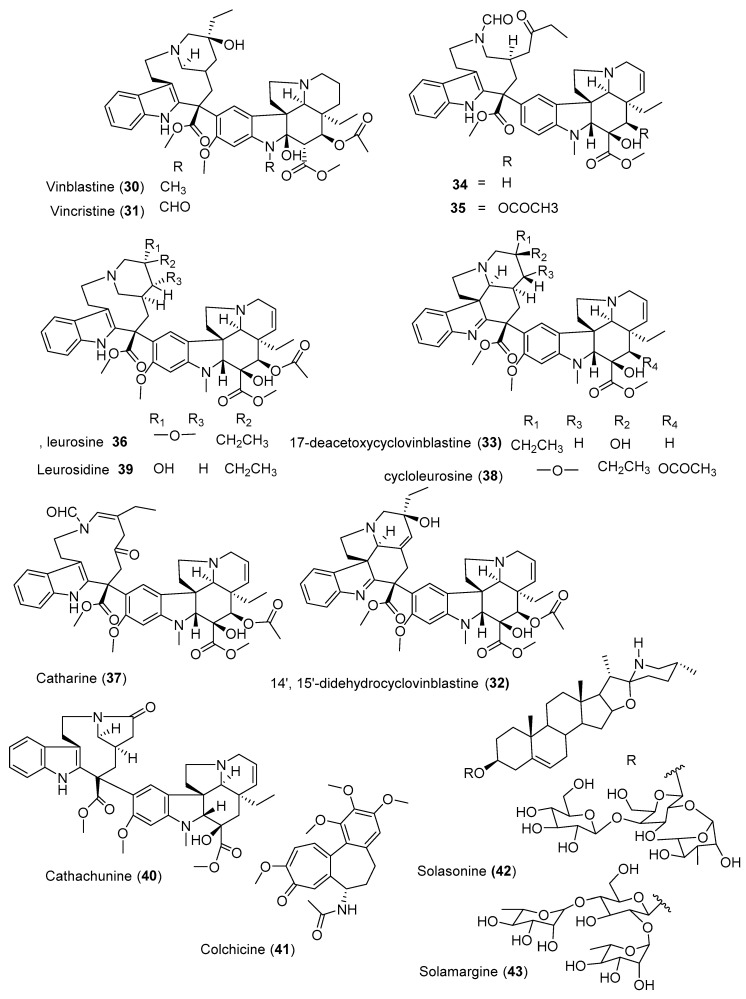
Structures of anticancer alkaloids reported from plants present in Ethiopia.

Vinblastine (**30**) and vincristine (**31**) (Figure 4) are one of the most effective bis-indole vinca alkaloids as anticancer drugs, isolated from the leaves of *Catharanthus roseus* [84]. This is one of the most precious anticancer plants indigenous to Madagascar. Previously, approximately 30 bis-indole alkaloids and over 60 monomeric indole alkaloids have been isolated from the aerial parts and roots of *C. roseus* [85,86]. Wang et al. [87] isolated three new cytotoxic dimeric indole alkaloids (**32**–**34**) along with other five known compounds from the whole plant of *C. roseus* collected from China (Table 4). Among the isolated compounds, leurosine (**36**) showed the most potent cytotoxic activity with IC_50_ value of 0.73 ± 0.06 µM. Furthermore, the isolated three new compounds (**32**–**34**) also showed potent cytotoxicity against triple-negative breast cancer (MDA-MB-231) cell line with IC_50_ values ranging from 0.97 ± 0.07 µM to 7.93 ± 0.42 µM. Another alkaloid, cathachunine (**40**), also showed a promising cytotoxic activity against HL-60 by inducing an intrinsic apoptotic pathway [88]. On the other hand, the monoterpenoid indole alkaloids vindoline and catharanthine, isolated from Malaysian *V. roseus*, showed weak cytotoxic activity against HCT 116 [89]. Furthermore, colchicine (**41**), isolated from the seeds of *Gloriosa superba*, demonstrated moderate activity against six human cancer cell lines (A549, MCF-7, MDA-MB231, PANC-1, HCT116, and SiHa) [90].

### 3.4. Steroids and Lignans

**Figure 5:**
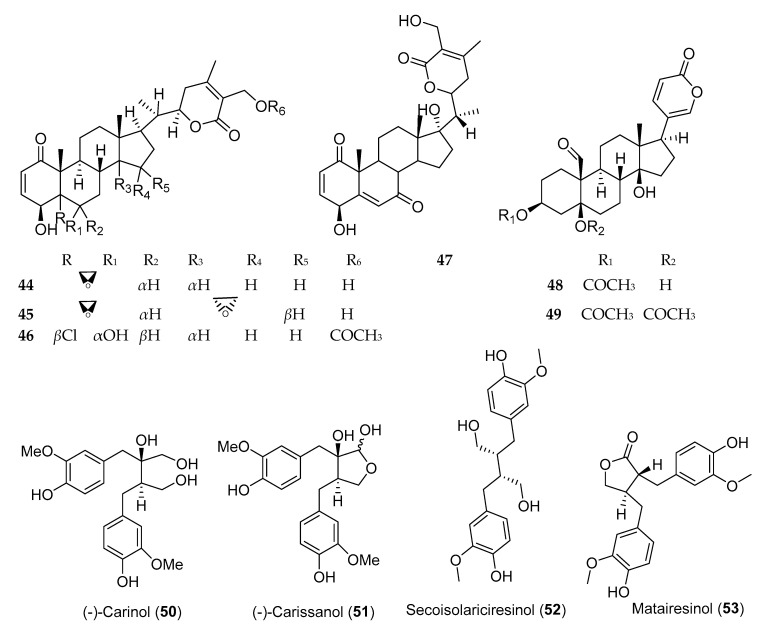
Structures of anticancer steroids and lignans reported from plants available in Ethiopia.

Steroids and lignans, in addition to other phytochemicals, are common secondary metabolites reported from Ethiopian plants. Evidence and epidemiological studies suggest that phytosterols and lignans are protective against a wide range of diseases and possess anticancer activity [93]. Withanolides are cytotoxic steroidal lactones, reported from various plants of the family Solanaceae [94], of which withaferine-A (**44**) and 5*β*,6*β*,14*α*,15*α*-diepoxy-4*β*,27-dihydroxy-1-oxowitha-2,24-dienolide (**45**) (Figure 5), isolated from *Withania somnifera*, demonstrated anticancer activity against human lung cancer cell line (NCI-H460) with IC_50_ values of 0.45 ± 0.00 and 8.3 ± 0.21 µg/mL, respectively [94]. Several buffadinolides, cardiac glycosides with steroidal nucleus, including berscillogenin, 3-epiberscillogenin, and bersenogenin [95]; hellebrigenin 3-acetate (**48**); and hellebrigenin 3,5-diacetate (**49**) [96] isolated from *Bersama abyssinica* collected from Ethiopia, demonstrated cytotoxic activities. *β*-Sitosterol-3-*O*-glucoside, a phytosterol from *Prunus Africana*, exhibited poor anticancer activity against three cell lines (Table 5).

Lignans and isoflavonoids are the major classes of phytoestrogens [97] which showed potential anticancer activity against various cells. Three lignans, namely, (−)-carinol (**50**), (−)-carissanol (**51**), and (−)-nortrachelogenin, isolated from *Carissa spinarum*,were found to be cytotoxic against A549, MCF-7, and WI-38 cell lines. Among these, (−)-carinol (i.e., a compound with butanediol structure) showed more potent cytotoxic activity against these three cell lines with IC_50_ value of 1 µg/mL, as compared to (−)-carissanol and (−)-nortrachelogenin [98]. Secoisolariciresinol (**52**) and matairesino (**53**), two lignans isolated from *Linum usitatissimum*, exhibited cytotoxicity against MCF-7 cells with IC_50_ values of 10 and 1 µM, respectively [99].

## 4. Preclinical, In Vivo, and Clinical Studies on Ethiopian Anticancer Plants

Preclinical studies generate data on the efficacy, safety, and pharmacokinetic properties of lead compounds, which will later be used to select better molecules for clinical trials. Assessment of the findings of preclinical in vivo animal studies supports the traditional use of plants to manage cancer in Ethiopia (Table 6). Despite the preclinical efficacy data, there are no clinically significant anticancer agents isolated from traditionally used Ethiopian plants. Moreover, there are also no clinical trials conducted on anticancer plants that are collected from Ethiopia. Among reviewed phytochemicals only ursolic acid, secoisolariciresinol (**52**), and colchicines (**41**), isolated from plants collected elsewhere, were considered further for clinical trial. 

## 5. Conclusions

Despite the traditional use of various Ethiopian plants for the treatment of cancer by herbal medicine practitioners for many decades, only a few active anticancer crude extracts, herbal preparations, and pure compounds were tested and so far no clinical trial was conducted on them. In this review, an attempt has been made to document antiproliferative, antitumor, and cytotoxic natural products small molecules isolated from medicinal plants that are traditionally used to treat cancer in Ethiopia. However, among the reported active compounds, only few have been isolated from plants that are originated and collected from Ethiopian geographic location, despite their wider presence and traditional claim at home. The majority of compounds reported in this review are isolated from plants (corresponding to Ethiopian species) that were collected from different regions of the world. However, the comprehensive list of active compounds (IC_50_ and ED_50_ values) provided in this review will help to identify the most potent source(s) of these compounds, as bioactive marker(s), of local flora. Based on the higher frequency of citation *Croton macrostachyus*, *Jatropha curcas*, *Plumbago zeylanica*, and *Vernonia hymenolepsis* are potential candidates for follow-up bioassay guided investigations. Furthermore, plants with reported antiproliferative compounds such as *Podocarpus falcatus*, *Linum usitatissimum*, and *Zehneria scabra* should also be examined for additional cytotoxic compounds and evaluated against a battery of cancer cell lines.

Generally, the ecological variation has a huge impact on the biosynthesis, yield of active constituent and biological potency of secondary metabolites produced by plants of similar species from different geographical regions. Thus, Ethiopian anticancer plants might have novel active constituents to fight cancer, based on traditional medical use, than those collected from other regions due to their unique geographical location and inherent climatic condition of the diverse landscape. Unfortunately, these valuable plant resources are disappearing rapidly due to climate change, rapid urbanization, agricultural land expansion, and artificial deforestation; therefore, Ethiopian flora is facing a great challenge, and thus it is high time to examine the anticancer plants systematically with the aim to carry out chemical and biological invesigations, as well as clinical trials on promising anticancer plant extracts based on ethnopharmacological knowledge.

## Figures and Tables

**Table 1:** General traditional use of selected Ethiopian medicinal plants.

Botanical Name (Family)	Illnesses/Symptoms Claimed to Be Treated Traditionally
*Bersama abyssinica* Fresen. (Melianthaceae)	Antispasmodic [19]; tumor [20]
*Carissa spinarum* (Apocynaceae)	Skin cancer [21]
*Catharanthus roseus* (L.) G. Don (Apocynaceae)	Cancer, liver infection, Wound, rheumatism [22]
*Centella asiatica* (L.) Urb. (Apiaceae)	Genital infection [23]; gastritis, evil eye, swelling [24]; Throat cancer [21]
*Croton macrostachyus* Hochst. Ex Delile (Euphorbiaceae)	Stomach ache, typhoid, worm expulsion, wounds, malaria [25]; wounds, malaria and gonorrhea [26]; tumor [27]; skin cancer, wound, ring worm [28]; cancer [29]
*Cucumis prophetarum* (Cucurbitaceae)	Skin cancer, cough, stomach-ache, diarrhoea [30]; wound, swollen body part [7]
*Ekebergia capensis* Sparrm. (Meliaceae)	Weight loss in children, stabbing pain, bovine tuberculosis [29]; cancer [6]
*Euphorbia tirucalli* L. (Euphorbiaceae)	Tumors [27]; wart, wounds [31]
*Ferula communis* L. (Apiaceae)	Gonorrhea [32]; Lung cancer [33]
*Gloriosa superba* (Colchicaceae)	Snake bite, impotence, stomach-ache [34]; tumors [35]
*Jatropha curcas* L. (Euphorbiaceae)	Abdominal pain [36]; rabies [25]; tumor [27,37]
*Juncus effusus* L. (Juncaceae)	Wound, stomach ache, bleeding after delivery, muscle cramps, tumors [27]
*Kniphofia foliosa* Hochst (Asphodelaceae)	Cervical cancer [21]
*Lagenaria siceraria* (Molina) Standl. (Cucurbitaceae)	Diarrhea, vomiting [38]; gonorrhea [39]; wound [25]; cough, cancer [28]
*Linum usitatissimum* (Linaceae)	Gastritis [40,41]
*Maytenus senegalensis* (Celastraceae)	Stomach-ache [42]; snake bite, tonsillitis, diarrhoea [43]; tumors [20]
*Olea europaea* subsp. Cuspidate (Wall. ex. G. Don) Cif. (Oleaceae)	Stomach problems, malaria, dysentery [44]; Eye disease [45]; wound [46]; brain tumor [47]
*Plumbago zeylanica* L. (Plumbaginaceae)	Cancer [26]; external body swelling, internal cancer, bone cancer [7]; cancer, cough, snake bite, swelling [31]
*Podocarpus falcatus* (Podocarpaceae)	Cancer [34]; amoeba, gastritis [6]; rabies [48]
*Premna schimperi* Engl. (Verbenaceae)	Antiseptic [49]; cancer [35]
*Prunus africana* (Hook.f.) Kalkman (Rosaceae)	Breast cancer [21]; benign prostatic hyperplasia, prostate gland hypertrophy [26]
*Ricinus communis* L. (Euphorbiaceae)	Rabies [48]; dysentery [50]; stomach ache [34,51]; Liver disease [52]; tooth ache [31]; breast cancer [28]
*Solanum nigrum* (Solanaceae)	Painful and expanding swelling on finger [7]; cancer [27]
*Vernonia amygdalina* Delile (Asteraceae)	Tonsillitis [34]; cancer [6]
*Vernonia hymenolepis* A. Rich. (Asteraceae)	Tumor [6,40,41]
*Withania somnifera* (Solanaceae)	Snake bite [53]; chest pain [54]; cancer [27]
*Zehneria scabra* (L.F. Sond) (Cucurbitaceae)	Fever, head ache [55]; tumor [56]; eye disease, wart [45]

**Table 3:** Phenolic compounds isolated from medicinal plants that are traditionally used to treat cancer in Ethiopia.

Plant	Class of Compounds	Cell Lines	IC_50_	Pharmacology	Isolated Active Compounds	Reference
*Maytenus senegalensis* (Celastraceae)	Phenolic	L5178Y	10 µg/mL (100% inhibition)	-	(−) Epigallocathechin (**25**)	[79]
*Juncus effusus* L. (Juncaceae)	Phenanthrenes	MCF-7	10.87 ± 0.82 µM	-	5-(1-Methoxyethyl)-1-methyl-phenanthren-2,7-diol (**26**)	[80]
			26.68 ± 2.95 µM		Effususol A (**27**)	
		HepG-2	23.90 ± 3.32 µM		Effusol	
		SHSY-5Y	22.83 ± 0.98 µM		Dehydroeffusol	
		HepG-2	23.13 ± 1.79 µM			
		SMMC-7721	25.35 ± 2.08 µM		Dehydroeffusal	
		HepG-2	12.43 ± 0.41 µM			
		Hela	13.07 ± 2.56 µM			
		HepG-2	26.04 ± 4.49 µM		5-Hydroxymethyl-1-methylphenanthrene-2,7-diol	
		Hela	16.35 ± 6.04 µM			
			29.63 ± 0.67 µM		2,7-Dihydroxy-1,8-dimethyl-5-vinyl-9,10-dihydrophenanthrene and juncusol	
		HepG-2	16.45 ± 1.12 µM		Dehydrojuncusol	
		Hela	15.17 ± 2.47 µM		1-Methylpyrene-2,7-diol	
		MCF-7	27.10 ± 1.17 µM			
	9,10-Dihydrophenanthrene	HT22	100 µM	Caspase-3-mediated cytotoxicity	Effususol A (**27**)	[81]
*Plumbago zeylanica*	Naphthoquinones	A549	10.3 µM	Apoptosis	Plumbagin (**28**)	[82]
		H292	7.3 µM			
		H460	6.1 µM			
		Panc-1	2.1 µM			[83]
Kniphofia foliosa Hochst*	Phenylanthraquinones	B16	3.3 ± 0.39 µM	Necrotic cell death	Knipholone (**29**)	[15]
		RAW 264.7	1.6 ± 0.25 µM			
		U937	0.5 ± 0.05 µM			
		THP-1	0.9 ± 0.09 µM			

Cell lines: SMMC-7721 = Human hepatocarcinoma, L5178Y = Mouse lymphoma, SHSY-5Y = human neuroblastoma, MCF-7 = Human breast adenocarcinoma, SMMC-7721 = Human hepatocarcinoma, HepG2 = Liver hepatocarcinoma, Hela = Human cervical cancer, HT22 = mouse hippocampal neuronal, B16 = mouse melanoma, RAW 264.7 = mouse macrophage tumor, THP-1 = human acute monocytic leukaemic, U937 = promonocytic leukaemic;, IC_50_ = Concentration that inhibited cell proliferation by 50%. * Plant material collected from Ethiopia.

**Table 4:** Alkaloids isolated from medicinal plants that are traditionally used to treat cancer in Ethiopia.

Plants	Class of Compounds	Cell Lines	IC_50_ Values	Pharmacology	Isolated Active Compounds	Reference
*Catharanthus roseus* (L.) G.Don (Apocynaceae)	Bisindole alkaloid	SH-SY5Y	0.1 µM	Mitotic arest and apoptosis	Vincristine (**31**)	[91]
		MDA-MB-231	0.67 ± 0.03 nM		Vinblastine (**30**)	[87]
	Indole alkaloids		0.97 ± 0.07 µM	-	14′,15′-Didehydrocyclovinblastine (**32**)	
			7.93 ± 0.42 µM		17-Deacetoxycyclovinblastine (**33**)	
			3.55 ± 0.19 µM		17–Deacetoxyvinamidine (**34**)	
			10.67 ± 0.63 µM		Vinamidine (**35**)	
			0.73 ± 0.06 µM		Leurosine (**36**)	
			8.59 ± 0.51 µM		Catharine (**37**)	
			1.11 ± 0.07 µM		Cycloleurosine (**38**)	
			4.26 ± 0.23 µM		Leurosidine (**39**)	
		HCT 116	>200 µg/mL		Vindoline	[89]
			60 µg/mL		Catharanthine	
	Bisindole alkaloid	HL-60	9.1 ± 0.7 µM	Induction of apoptosis via an intrinsic pathway	Cathachunine (**40**)	[88]
*Gloriosa superba* (Colchicaceae)	Alkaloid	A-549 and MDA-MB-231	60 nM	G2/M phase arrest	Colchicine (**41**)	[90]
*Solanum nigrum* (Solanaceae)	Steroidal glycoalkaloids	MGC-803	5.2 µg/mL	Apoptosis	Solasonine (**42**)	[92]
			26.5 µg/mL		*β*1-Solasonine	
			8.77 µg/mL		Solamargine (**43**)	
			20.1 µg/mL		Solanigroside P	

Cell lines: MDA MB 231 = Triple-negative breast cancer, SW480 = Human colorectal, HCT116 = Human colorectal carcinoma, HL60 = Human promyelocytic leukemia, MCF-7 = Human breast adenocarcinoma, SMMC-7721 = Human hepatocarcinoma, A-549 = Human lung adenocarcinoma, MGC-803 = Human gastric cancer. IC_50_ = Concentration that inhibited cell proliferation by 50%.

**Table 5:** Steroidal and Lignan compounds isolated from medicinal plants that are traditionally used to treat cancer in Ethiopia.

Plant	Class of Compounds	Cell Lines	IC_50_	Isolated Active Compounds	Reference
*Prunus africana* (Hook.f.) Kalkman (Rosaceae)	Steroids	HEK293	937 µg/mL	*β*-Sitosterol-3-*O*-glucoside	[93]
		HepG2	251 µg/mL		
		Caco-2	54 µg/mL		
*Withania somnifera* (Solanaceae)	Steroidal lactone	NCI-H460	0.45 ± 0.00 µg/mL	Withaferin A (**44**)	[94]
			8.3 ± 0.12 µg/mL	5*β*,6*β*,14*α*,15*α*-Diepoxy-4*β*,27-dihydroxy-1-oxowitha-2,24-dienolide (**45**)	
			95.6 ± 2.60 µg/mL	27-Acetoxy-4*β*,6*α*-dihydroxy-5*β*-chloro-1-oxowitha-2,24-dienolide (**46**)	
	Withasteroid	MCF-7 and WRL-68	1.0 µg/mL	5,6-De-epoxy-5-en-7-one-17-hydroxy withaferin A (**47**)	[100]
		Caco-2	3.4 µg/mL		
		PC-3	7.4 µg/mL		
*Bersama abyssinica* Fresen.* (Melianthaceae)	Steroids (bufadienolide)	KB	0.028 µg/mL (ED_50_)	Berscillogenin	[95]
			0.62 µg/mL (ED_50_)	3-Epiberscillogenin	
			0.0046 µg/mL (ED_50_)	Bersenogenin	
			10^−7^ µg/mL (ED_50_)	Hellebrigenin 3-acetate (**48**)	[96]
			10^−3^ µg/mL (ED_50_)	Hellebrigenin 3,5-diacetate (**49**)	
*Carissa spinarum* (Apocynaceae)	Lignans	A549	<1 µg/mL	(−)-Carinol (**50**)	[98]
		MCF-7			
		WI-38			
		A549	11.0 µg/mL	(−)-Carissanol (**51**)	
		MCF-7	17.4 µg/mL		
		WI-38	6.2 µg/mL		
		A549	29.0 µg/mL	(−)-Nortrachelogenin	
		MCF-7	88.3 µg/mL		
		WI-38	>100 µg/mL		
*Linum usitatissimum* (Linaceae)	Lignans	MCF-7	1 × 10^−5^ mol/L	Secoisolariciresinol (**52**)	[99]
			1 × 10^−6^ M	Matairesinol (**53**)	

Cell lines: HEK293 = Human embryonic kidney, HepG2 = Liver hepatocarcinoma, Caco-2 = Human colon carcinoma, NCI-H460 = Human large-cell lung carcinoma, MCF-7 = Human breast adenocarcinoma, WRL-68 = human hepatic, PC-3 = Human prostate cancer, KB = Human mouth epidermal carcinoma, MGC-803 = Human gastric cancer, A-549 = Human lung adenocarcinoma, WI-38 = Normal human embryonic, IC_50_ = Concentration that inhibited cell proliferation by 50%. ED_50_ = Effective dose for 50% of the population * Plant material collected from Ethiopia.

**Table 6:** Animal efficacy studies, clinical trials, and/or clinically approved agents among Ethiopian anticancer plants/compounds.

Plants	Crude Extract	Isolated Compounds	In Vivo Studies	Clinical Trials (Status)	Clinically Approved for
*Bersama abyssinica*		Hellebrigenin 3-acetate (**48**)	Significantly inhibits Walker intramuscular carcinosarcoma 256 in rats [96]	-	-
*Catharanthus roseus*	Ethanolic extract		Significantly increased the life span and decreased the tumor volume in Ehrlich ascites carcinoma-bearing mice [101]	-	-
		Vincristine (**31**)	-	-	Childhood leukaemia, Hodgkin’s disease and acute panmyelosis [102]
		Vinblastine (**30**)	-	-	Lymphosarcoma, choriocarcinoma, neuroblastoma and lymphocytic leukemia [103]
*Euphorbia tirucalli*	Hydroalcoholic extract		Significantly enhanced survival and reduced tumor growth in Ehrlich ascites tumor-bearing mice [104]	-	-
	Latex		Significantly reduced tumor growth and cachexia in Walker 256 tumor-bearing rats [105]	-	-
*Gloriosa superba*	Ethanolic crude extract		Significantly reduced tumor growth in combination with gemcitabine in a murine model of pancreatic adenocarcinoma [106]	-	-
		Colchicine (**41**)	-	Phase II for castrate resistant prostate cancer (Withdrawn due to funding) [107]	-
*Jatropha curcas*	Methanolic fractions		Showed significant anti-metastatic and antiprolifertaive activity in C57BL/6 mice [108]	-	-
*Linum usitatissimum*		Secoisolariciresinol (**52**)	-	Phase II (Completed) [109]	-
*Prunus Africana*	Ethanol extract		Showed significant reduction in prostate cancer incidence in mice [110]	-	
		Ursolic Acid	-	Early Phase I [111]	-
*Plumbago zeylanica* L.		Plumbagin	Significantly inhibits squamous cell carcinomas in FVB/N mice [112]		
*Ricinus communis*	Fruit extract		Significantly reduced tumor volume in 4T1 syngeneic mouse model [113]	-	-
*Solanum nigrum*	Crude polysaccharides		Significant growth inhibition in cervical cancer tumor-bearing mice [114]	-	-
	Aqueous extract		Significantly inhibits early hepatocarcinogenesis [115]	-	-
*Vernonia amygdalina*	Aqueous crude extract		Increase efficacies and optimizes treatment outcomes when given with paclitaxel in athymic mice [116]	_	-
*Vernonia hymenolepis*		Vernolepin	Significantly inhibited intramuscular carcinosarcoma in walker tumor bearing rats [117]	-	-
*Withania somnifera*	Aqueous extract		Decreased tumor volume in orthotopic glioma allograft rat model [118]	-	-
	Ethanolic extract		Significantly improve colon cancer treatment in mice [119]	-	-
		Withaferin A	Significantly inhibited HepG2-xenografts and diethylnitrosamine (DEN)-induced-hepatocellular carcinoma (HCC) in C57BL/6 mice [120]	-	-
